# QuickStats

**Published:** 2013-08-30

**Authors:** Jiaquan Xu

**Figure:**
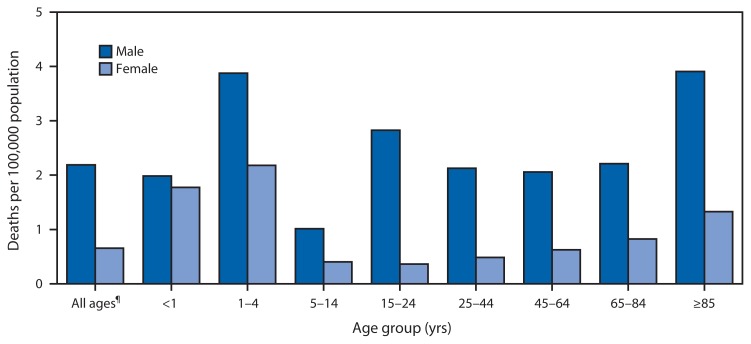
Average Annual Death Rates from Drowning,*^†^ by Sex and Age Group — United States,^§^ 1999–2010 * Drowning from all intents (unintentional, homicide, suicide, and undetermined) as the underlying cause of death, coded as W65–74, X71, X92, and Y21, in the *International Classification of Diseases, 10th Revision*. This excludes accidents to watercraft causing drowning and submersion (V90) and water-transport–related drowning and submersion without accident to watercraft (V92). ^†^ Per 100,000 population, based on 12-year annual average. ^§^ U.S. residents only. ^¶^ Includes decedents whose ages were not reported.

During 1999–2010, a total of 49,762 deaths from drowning occurred in the United States, an average of 4,147 deaths per year. The average annual death rate from drowning for males (2.2 per 100,000 population) was more than three times that for females (0.7). The death rate for males was highest among those aged 1–4 years and ≥85 years (both 3.9 per 100,000 population). For females, the highest rates were among those aged 1–4 years (2.2) and <1 year (1.8).

**Source:** National Vital Statistics System. Mortality public use data files, 1999–2010. Available at http://www.cdc.gov/nchs/data_access/vitalstatsonline.htm.

